# Insomnia drug therapy in COVID-19 patients; a letter to editor

**DOI:** 10.22088/cjim.11.0.583

**Published:** 2020

**Authors:** Ali Rismanbaf

**Affiliations:** 1 Department of Clinical Pharmacy and Pharmacy Practice, School of Pharmacy and Pharmaceutical Sciences, Isfahan University of Medical Sciences, Isfahan, Iran

**Dear Sir,**

Coronavirus disease 2019 (COVID-19) is a highly contagious disease caused by the newly emerging SARS-CoV-2 virus that was first reported in December 2019 and was announced as a pandemic disease on March 11^th^, according to a World Health Organization (WHO) warning (1). COVID-19 requires treatment in isolation because of its transmission through respiratory droplets and close contact. It has been reported that many patients developed sleep disturbances due to isolation treatment or medication use and need to prescribe hypnotic medications (2). Also, some patients may have taken hypnotic medications before developing COVID-19 and may already be dependent on these medications, and now, with the onset of COVID-19 and the addition of new drugs for its treatment, they need to change their hypnotic medications.

Three important factors that may influence the choice of the appropriate hypnotic medication in COVID-19 patients include the interactions of hypnotic medications with common medications in the treatment of COVID-19, respiratory depression caused by hypnotic medications, and the effect of liver damage, caused by SARS-CoV-2 or COVID-19 drug therapy, on hypnotic medications metabolism (3). The medications that are mainly used to improve sleep quality include Benzodiazepines (Estazolam, Flurazepam, Quazepam, Temazepam, and Triazolam), Nonbenzodiazepines (Zolpidem, Eszopiclone, and Zaleplon), Orexin Receptor Antagonists (Suvorexant) and Antidepressants (Doxepin). According to Up-to-date and Lexi-Drugs drug information programs, Kaletra (Ritonavir/Lopinavir) and Atazanavir have drug interactions with Suvorexant, Zolpidem, Eszopiclone, Estazolam, and Triazolam, and there is no significant interaction between Chloroquine and Ribavirin with hypnotic medications. Also, the metabolism of Doxepin, Suvorexant, Zolpidem, Eszopiclone, and Zaleplon in liver damage can be affected and better not to prescribe. On the other hand, the risk of respiratory infection following Doxepin, Suvorexant, and Zolpidem and the risk of viral infection following Eszopiclone, and influenza following Zolpidem have been reported. Also among the hypnotic medications, most respiratory complications are seen with Doxepin, Suvorexant, Zolpidem, Zaleplon, and Flurazepam.

As a result, best hypnotic medications to administer in COVID-19 patients with insomnia are Quazepam and Temazepam that they have the least drug interactions with COVID-19 drugs, liver damage has the least effect on their metabolism, and their respiratory complications are less than other sleep medications. However, being aware of respiratory depression and careful monitoring of COVID-19 patients' respiratory function is recommended when prescribing hypnotics, even Quazepam and Temazepam.
